# Planning for return to work during the first year after breast cancer metastasis: A Swedish cohort study

**DOI:** 10.1002/cam4.5752

**Published:** 2023-03-06

**Authors:** Aina Johnsson, Narsis A. Kiani, Sofie A. M. Gernaat, Ulla Wilking, Ivan Shabo, Elham Hedayati

**Affiliations:** ^1^ Department of Oncology‐Pathology Karolinska Institute Stockholm Sweden; ^2^ Division of Clinical Epidemiology, Department of Medicine Karolinska Institute Stockholm Sweden; ^3^ Breast Cancer Centre, Cancer Theme Karolinska University Hospital, Karolinska CCC Stockholm Sweden; ^4^ Department of Molecular Medicine and Surgery Karolinska Institute Stockholm Sweden

**Keywords:** advanced breast cancer, return to work, survival, tumour burden, work ability

## Abstract

**Background:**

Planning for return to work (RTW) is relevant among sub‐groups of metastatic breast cancer (mBC) survivors. RTW and protective factors for RTW in patients with mBC were determined.

**Methods:**

Patients with mBC, ages 18–63 years, were identified in Swedish registers, and data were collected starting 1 year before their mBC diagnosis. The prevalence of working net days (WNDs) (>90 and >180) during the year after mBC diagnosis (y1) was determined. Factors associated with RTW were assessed using regression analysis. The impact of contemporary oncological treatment of mBC on RTW and 5‐year mBC‐specific survival was compared between those diagnosed in 1997–2002 and 2003–2011.

**Results:**

Of 490 patients, 239 (48.8%) and 189 (36.8%) had >90 and >180 WNDs, respectively, during y1. Adjusted odds ratios (AORs) of WNDs >90 or >180 during y1 were significantly higher for patients with age ≤50 years (AOR_180_ = 1.54), synchronous metastasis (AOR_90_ = 1.68, AOR_180_ = 1.67), metastasis within 24 months (AOR_180_ = 1.51), soft tissue, visceral, brain as first metastatic site (AOR_90_ = 1.47) and sickness absence <90 net days in the year before mBC diagnosis, suggesting limited comorbidities (AOR_90_ = 1.28, AOR_180_ = 2.00), respectively. Mean (standard deviation) WNDs were 134.9 (140.1) and 161.3 (152.4) for patients diagnosed with mBC in 1997–2002 and 2003–2011, respectively (*p* = 0.046). Median (standard error) mBC‐specific survivals were 41.0 (2.5) and 62.0 (9.6) months for patients diagnosed with mBC in 1997–2002 and 2003–2011, respectively (*p* < 0.001).

**Conclusions:**

RTW of more than 180 WNDs was associated with younger age, early development of metastases and limited comorbidities during the year before the diagnosis of mBC. Patients diagnosed with mBC in 2003 or later had more WNDs and better survival than those diagnosed earlier.

## Graphical abstract

About half of the women with stage 4 breast cancer return to work one after diagnosis.

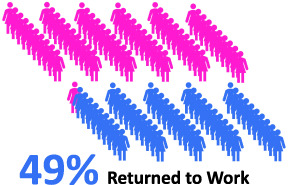

## 1 INTRODUCTION

Globally, breast cancer (BC) is the most prevalent malignant disease in women.^1^ Up to 10% of patients with BC present with metastatic stage IV breast cancer (mBC) and are, thus, considered to have synchronous metastasis.^2^, ^3^, ^4^, ^5^ In addition, up to 30% of patients with early‐stage (ductal carcinoma in situ and stage I–III) breast cancer (eBC) develop metastases later and are, thus, considered to have metachronous metastasis.^6^, ^7^ During the late 1990s and the beginning of the 2000s, significant progress was made in the treatment of mBC.^8^, ^9^, ^10^ In April 2002, the Swedish Breast Cancer Group updated its guideline for mBC due to the introduction of more effective oncological agents and advances in supportive care have increased overall survival and health,^11^, ^12^, ^13^, ^14^ transforming the mBC into a chronic condition. Since 2003, the treatment recommendations still apply and are part of contemporary treatment recommended by the Swedish Breast Cancer Group.^14^ Median survival for patients with mBC appears to have improved over time, a trend attributed to the availability of new, more effective agents, including taxanes, aromatase inhibitors and trastuzumab.^15^, ^16^, ^17^, ^18^, ^19^ The current median overall survival among patients diagnosed with mBC in Sweden is 29.8 months from diagnosis, the 5‐year survival is 30% and the 10‐year survival is 14%.^12^

On the molecular level, mBC is a heterogeneous disease due to alterations acquired during the disease evolution from its early to late stages. The tumour biology of mBC is not always representative of that of eBC.^20^, ^21^ The molecular features include activation of human epidermal growth factor receptor 2 (HER2, encoded by ERBB2), activation of hormone receptors (oestrogen receptor and progesterone receptor) and BRCA mutations. Treatment strategies to shrink or slow cancer growth differ according to molecular subtype. Management of mBC is multidisciplinary; systemic therapy is the first therapeutic choice in mBC, and locoregional therapy (e.g. surgery, radiotherapy) can be added in specific situations (e.g. primary metastatic disease, symptomatic bone or brain metastases). Contemporary systemic therapies include endocrine therapy for ER/PR‐positive disease, chemotherapy, anti‐HER2 therapy for HER2‐positive disease, bone‐stabilising agents, poly (ADP‐ribose) polymerase inhibitors for germline BRCA1/2 mutations and, quite recently, immunotherapy for hormone receptor‐negative and HER2‐negative disease.^14^, ^22^ Next to prolongation of survival, therapeutic goals in mBC are maintenance of the quality of life and palliation of symptoms. Therapy concepts, the duration of therapy and the amount of treatment lines are usually more individualised in mBC since patients differ regarding preferences, pre‐treatments, disease response and residual side effects from previous therapies. Tumour biology is essential together with the duration of response to prior therapies and tumour burden with associated symptoms.^22^

In Sweden, half of the women living with mBC are of working age, defined as 18 and 65 years old.^23^, ^24^ As women with mBC can live longer with their chronic disease, planning for a full or part‐time return to work (RTW) is relevant and realistic for a subgroup of patients.^4^ Furthermore, being employed is, for most persons, an essential part of well‐being^25^, ^26^ and the feeling of normalcy.^27^ Other reasons for considering work important are financial and insurance issuers, the importance of staying busy and the desire to support themselves and their family.^28^ In Sweden, employees have legal protection against dismissal in the event of illness.^29^ As far as possible, the employer must adopt the workplace/work tasks so that a person on sick leave can RTW.^30^, ^31^ Anyone who, due to illness, lacks work ability can receive compensation via the public health insurance system. The SSIA administers the public health insurance system and is financed via employer contributions. The public health insurance system covers everyone who resides in Sweden. Compensation for the loss of income of up to 80% of the salary can be paid. In case of serious illness, where mBC is counted, the employee has the right to keep his sickness benefits if returning to work with the former employer is not possible.^30^

Among Swedish women diagnosed with eBC, higher‐stage of eBC, chemotherapy and long‐term sickness absence (SA)/disability pension (DP) before eBC diagnosis constitute the most significant risk for long‐term SA/DP after eBC diagnosis.^32^, ^33^, ^34^ Regarding the importance of other socio‐demographic factors, the results are divergent.^32^, ^33^, ^34^, ^35^, ^36^ Long‐term SA before eBC diagnosis, DP, or severe adverse sequelae after eBC treatment contributed to a high prevalence of long‐term SA among Swedish women who later received an mBC relapse.^37^ Moreover, SA or DP during the year before the mBC diagnosis contributes to a high prevalence of sickness benefit utilisation after the mBC diagnosis.^38^ Studies from the USA have shown that women work less after an mBC diagnosis than before, have lower performance scores, higher symptom burden/ pain and/other symptoms, are older, further from initial BC diagnosis and mBC, have high rates of life interference due to mBC, and belonging to ethnic minority groups are associated with lower employment rates.^27^, ^28^, ^39^, ^40^ Self‐employed, short‐term SA, receiving hormonal treatment, and decreasing symptoms are associated with continuing to work.^27^, ^39^ Thus, limited research has explored the ability to RTW among women living with mBC. Furthermore, up to date, according to our knowledge, there were no relevant publications in PubMed aiming to study the association between tumour and treatment‐related factors of mBC and RTW.

The primary aim of this study was to determine the extent of RTW in women of working age in Sweden with the survival of at least 1 year after the diagnosis of mBC and to identify the demographic and tumour‐related factors that influenced RTW during this year. The secondary aim was to determine the impact of contemporary oncological treatment of mBC on RTW by comparing those diagnosed with mBC between 1997 and 2002 to those diagnosed between 2003 and 2011. A third aim was to compare the 5‐year mBC‐specific survival of those diagnosed with mBC between 1997 and 2002 to those diagnosed between 2003 and 2011.

## 2 METHODS

This study was approved by the Regional Ethics Review Board at the Karolinska Institute (Dnr 2012/745‐31) and complied with the Declaration of Helsinki. As a result of Swedish legislation, patients included in national quality registers do not need to provide written informed consent for their data to be included in healthcare research, and they have been informed that their data has been included in registers and they can drop out of the registers at any time.

### 2.1 Data sources

Diagnostic data for the study were obtained from two Swedish national registers: (i) the Breast Cancer Registry (BCR), which included data for patients from the Stockholm‐Gotland healthcare region diagnosed with mBC from January 1997 through December 2007; and (ii) the National Quality Register for Breast Cancer (NKBC), which included data for patients from the Stockholm‐Gotland healthcare region diagnosed with mBC from January 2008 through December 2011.

The Swedish Social Insurance Agency (SSIA) Microdata for Analyses of Social Insurance (MiDAS) database was used to retrieve data about SA and DP from January 1996 through December 2014. The MiDAS database contains information from SSIA about all continuous sickness/disability benefits payments, which usually begin on day 15 of sick leave episodes since the first 14 days is compensated by the employer. SSIA compensates for the loss of income due to the inability. The compensation levels are 100%, 75%, 50%, or 25%, depending on the portion of scheduled working hours that cannot be performed. SA and DP net days are calculated by multiplying the level of benefit received (i.e. 100%, 75%, 50% or 25%) by the total number of SA or DP days. This categorisation can be reversed to calculate the RTW for any working hours not being compensated.

The guaranteed pension age in Sweden was 65 years old; early withdrawal was possible from 63 years old.

Data about the time of death were collected from the Swedish Cause of Death Register, maintained by the Swedish National Board of Health and Welfare. The Swedish Longitudinal Integrated Database for Health Insurance and Labour Market Studies (LISA) obtained data about marital status and the number of children. Data from these registers were linked together for each patient using the unique national identification number assigned to each resident in Sweden at birth or when establishing permanent residency.

### 2.2 Study population and cohorts

A population‐based cohort study was conducted, including all 1240 women in the Stockholm‐Gotland aged 18–63 years diagnosed with mBC from January 1, 1997, through December 31, 2011, and registered in the BCR or NKBC. Patients in this study had SA or DP data available from the MiDAS database 1 year before to 1 year after the mBC diagnosis. Patients were excluded if they died during the first year post‐mBC diagnosis (planning for long‐term RTW is not realistic) and had more than 180 net days of SA or had any DP during the year before mBC diagnosis (already left the labour market due to other reasons than mBC).

The calendar year of mBC diagnosis was used as a general proxy for determining the impact of contemporary oncological treatment of mBC in the study. Patients in this study were divided into two cohorts: (i) the historical cohort, including patients who were diagnosed with mBC from 1997 through 2002 and (ii) the current cohort, including patients who were diagnosed with mBC from 2003 through 2011, and which was after the introduction of current oncological therapy for mBC. The cut‐off for the calendar year 2003 was chosen as a proxy for contemporary oncological treatment of mBC since the Swedish Breast Cancer Group updated the national guidelines for mBC in April 2002. By January 2003, the recommendations were fully implemented in the Stockholm‐Gotland healthcare region, and full‐year data were available for this study.

### 2.3 Outcome measures

The primary outcome measures used for the study were RTW for more than 90 working net days (WNDs > 90) and for more than 180 WNDs (WND > 180), each during the year after mBC diagnosis.

Net working days were defined as the number of days without full‐time compensated SA multiplied by the percentage of cessation of compensation received (i.e. 25%, 50% or 100%) for each of those days. Net working days were then grouped into a minimum of 25% partial RTW (WND > 90) or a minimum of 50% partial RTW (WND > 180).

Metastatic BC‐specific survival was defined as the time (in months) between the date of first mBC diagnosis and the date of death or the end of follow‐up. For this study, data were collected for at least 5 years after mBC diagnosis or until December 31, 2016, whichever was later.

### 2.4 Covariates

We recorded and categorised each patient's age, marital status and calendar year at the initial mBC diagnosis. We grouped the metastasis‐free time interval by noting whether the mBC was synchronous or metachronous. We logged the number of distant metastases at the initial mBC diagnosis and the site of the first distant metastasis, which was based on International Classification of Diseases, Ninth Edition (ICD‐9) codes (Table S1). We also recorded SA net days during the year before mBC diagnosis.

### 2.5 Metastatic BC treatment before and after 2003

The Swedish Breast Cancer Group is responsible for an evidence‐based national guideline for BC, updated when there are substantial changes in evidence.^14^ Oncologists are obliged to follow their recommendations in Sweden. In April 2002, the Swedish Breast Cancer Group updated the national guidelines for mBC.

Since April 2002, the Swedish Breast Cancer Group recommended therapy strategy for postmenopausal women with hormone‐positive mBC has been aromatase inhibitors as the first choice, anastrozole or letrozole, replacing tamoxifen. The recommended treatment for premenopausal women with hormone‐positive mBC has been oophorectomy (medical, radiological or surgical) combined with tamoxifen instead of only tamoxifen.

Before April 2002, the recommended chemotherapeutic regiments, as first‐line and second‐line treatment of mBC, were anthracycline‐containing chemotherapy, FEC (5‐fluorouracil, epirubicin, cyclophosphamide) or non‐anthracycline‐containing chemotherapy CMF (cyclophosphamide, 5‐fluorouracil and methotrexate) both administered intravenously at 3‐week intervals. After April 2002, the recommended therapy strategy has been anthracycline‐containing chemotherapy and taxane‐containing regimens for first‐line and second‐line mBC treatment. Since April 2002, the recommended therapy strategy for patients with Her2 positive mBC has been trastuzumab treatment instead of no treatment option.

Palliative radiation therapy as locoregional therapy can be added in specific situations (e.g. symptomatic bone, lung, skin or brain metastases). It is an effective treatment for pain, dyspnoea, discomfort, ulceration, bleeding, malodour, seizure, nausea/vomiting and shrinkage of brain metastasis.

For patients with verified ER‐positive tumours, endocrine treatment is the first choice (except for aggressive relapses/especially in visceral organs, “visceral crisis”) as long as the patient has objective benefit from it and does not suffer unacceptable side effects. For chemotherapy, the same overall goal applies. Previous studies highlight the importance of long term, continuous treatment compared with short and intermittent treatment. Primarily, sequential treatment is given with one cytostatic at a time. Combination therapy may be considered in case of acutely threatening visceral metastasis.

### 2.6 Statistical methods

Univariable and multivariable logistic regression analyses were performed to estimate the crude odds ratios, adjusted odds ratios (AORs) and 95% confidence intervals (CIs) of the primary outcome variables for each demographic and clinical characteristic category. We did separate regression analyses for RTW for WND > 90 and WND > 180 during the year after mBC diagnosis. The adjusted models included age, calendar year of mBC diagnosis, SA net days in the year before mBC diagnosis (all as continuous variables), marital status and the number of distant metastatic sites at mBC diagnosis (as categorical variables).

The WNDs of patients during the year after being diagnosed with mBC for the intervals of 1997 to 2002 and 2003 to 2011 were each determined, then reported as means and standard deviations (SD) and compared. WNDs were also compared within other variable groups, including age at mBC diagnosis, metastasis‐free time interval and SA during the year before mBC diagnosis. The two‐sample test was used for these comparisons. The prevalence of patients within each 30‐day WNDs category (ranging from 0 to 30 WNDs to 331 to 360 WNDs) was also determined for each of the two diagnosis intervals and presented as histograms.

Survival rates specific to mBC were estimated using Kaplan–Meier curves, and both medians and standard errors (SE) were calculated for each of the two mBC‐diagnosis‐year cohorts. The log‐rank test was used to determine the statistical significance of differences in mBC‐specific survival rates between the two mBC‐diagnosis‐year groups. For all analyses, *p* < 0.05 was considered statistically significant. The statistical analyses were performed using SPSS, version 25, and MATLAB, version 2017b.

## 3 RESULTS

### 3.1 Patient characteristics

A total of 1240 patients fit the inclusion criteria of the study. Of these, 750 were excluded, 435 (35.1%) died during the first year after mBC diagnosis, and 315 (25.4%) had >180 net SA days or any DP during the year before mBC diagnosis. A total of 490 patients remained and comprised the study population. The median (range) age of this population was 51 (25–63) years, 436 (89.0%) of these patients had metachronous mBC, and the population was roughly equally distributed between the two mBC‐diagnosis‐year cohorts, 1997 to 2002 and 2003 to 2011 (Table 1).

**TABLE 1:** Demographic and clinical characteristics of 490 female patients^a^ with new metastatic breast cancer (mBC) diagnosis in Stockholm‐Gotland Region, Sweden, January 1, 1997 to December 31, 2011.

Characteristics	Patients
	*n* (%)
Total population	490 (100)
Age at mBC diagnosis, years	
≤50	348 (71)
>50	142 (29)
Marital status at mBC diagnosis	
Married/cohabiting	275 (56)
Not married/cohabiting	215 (44)
Number of children at mBC diagnosis	
0	99 (19)
1–2	296 (61)
3–6	95 (20)
Calendar year of mBC diagnosis	
1997–2002	237 (48)
2003–2011	253 (52)
Metastasis‐free time interval, months	
<6 (Synchronous^b^)	54 (11)
6–24	76 (16)
>24	360 (73)
Site of first distant metastasis	
Bone	241 (49)
Soft tissue, visceral and/or brain	227 (46)
Not defined^c^	22 (5)
Number of distant metastatic sites at mBC diagnosis	
1	373 (76)
>1	111 (23)
Not defined^c^	6 (1)
Sickness absence (SA) during year before mBC diagnosis, net days^d^	
0–30	356 (73)
31–90	77 (15)
91–180	57 (12)

^a^ Patients included those who were between 18 and 63 years old at the time of the diagnosis of mBC, lived at least 1 year after the diagnosis of mBC, had sickness absence (SA) or disability pension (DP) data available for the period 1 year before through 1 year after the diagnosis of mBC and did not have any DP or SA for more than 180 net days during the year before mBC diagnosis.

^b^ Synchronous is defined as primary mBC or the development of mBC less than 6 months after an early breast cancer diagnosis.

^c^ Could not be determined because of the multifocality of metastases.

^d^ Net days are calculated by multiplying the benefit received (i.e. 25%, 50%, 75% or 100%) by the total number of SA days benefit received.

### 3.2 Ability to RTW

During the first year after mBC diagnosis, 239 (48.8%) patients had a minimum of 25% partial RTW (WND > 90), and 189 (38.6%) patients had a minimum of 50% partial RTW (WND > 180) (Table 2).

**TABLE 2:** Crude and adjusted odds ratios (OR) for having returned to work (RTW) more than 90 net days (WND > 90) and more than 180 working net days (WND > 180) during the year after a diagnosis of metastatic breast cancer (mBC), by demographic and clinical characteristics, among 490 female patients^a^ in Stockholm‐Gotland healthcare region, Sweden, January 1, 1997, to December 31, 2011.

Variables	Return to work (RTW)					
	More than 90 WND			More than 180 WND		
	Patient with WND ≤ 90/WND > 90, *n*/*n*	Crude OR (95% CI)	Adjusted OR^b^ (95% CI)	Patient with WND ≤ 180/WND > 180, *n*/*n*	Crude OR (95% CI)	Adjusted OR^b^ (95% CI)
Total population	239/251			189/301		
Age at mBC diagnosis, years						
≤50	163/185	1.21 (0.92–1.60)	1.07 (0.87–1.77)	122/226	**1.42 (1.08–1.87)**	**1.54 (1.35–1.84)**
>50	76/66	1	1	67/75	1	1
Marital status at mBC diagnosis						
Married/cohabiting	136/139	1.06 (0.74–1.52)	1.01 (0.69–1.47)	106/169	0.10 (0.69–1.44)	0.97 (0.66–1.42)
Not married/cohabiting	103/112	1	1	83/132	1	1
Number of children at mBC diagnosis						
0	56/43	1	1	41/58	1	1
1–2	135/161	0.78 (0.45–1.38)	0.71 (0.39–1.28)	110/186	0.94 (0.53–1.67)	0.83 (0.45–1.52)
3–6	48/47	1.22 (0.77–1.94)	1.13 (0.69–1.84)	38/57	1.13 (0.70–1.81)	0.99 (0.60–1.63)
Metastasis‐free time interval, months						
<6 (Synchronous^c^)	38/16	**2.94 (1.68–5.16)**	**1.69 (1.09–2.62)**	31/23	**2.95 (1.77–4.92)**	**1.67 (1.04–2.68)**
6–24	42/34	1.02 (0.84–1.20)	**1.86 (1.05–2.71)**	34/42	1.79 (0.64–2.05)	**1.51 (1.24–2.34)**
>24	159/201	1	1	124/236	1	1
First site of distant metastasis						
Bone	115/126	1	1	93/148	1	1
Soft tissue, visceral, brain	108/119	1.00 (0.85–1.20)	**1.47 (1.05–2.05**)	86/141	1.02 (0.85–1.22)	1.11 (0.73–1.69)
Not defined^d^	16/6	‐	‐	10/11	‐	‐
Number of distant metastatic sites at mBC diagnosis						
1	190/183	**1.64 (1.07–2.50)**	1.52 (0.94–2.46)	150/223	**1.59 (1.01–2.51)**	1.40 (0.85–2.32)
>1	43/68	1	1	33/78	1	1
Not defined^d^	6/0	‐	‐	6/0	‐	‐
Sickness absence (SA) in year before mBC diagnosis, net days^e^						
0–90	222/211	**3.45 (2.75–4.08)**	**1.28 (1.04–2.40)**	133/118	**4.17 (2.64–4.11)**	**2.00 (1.52–2.72)**
91–180	17/40	1	1	56/183	1	1

*Note*: Bolded results were statistically significant.

Abbreviations: 95% CI, 95% confidence interval; mBC, metastatic breast cancer; OR, odds‐ratios; RTW, return to work, WND working net days.

^a^ Patients included those who were between 18 and 63 years old at the time of the diagnosis of mBC, lived at least 1 year after the diagnosis of mBC, had sickness absence (SA) or disability pension (DP) data available for the period 1 year before through 1 year after the diagnosis of mBC and did not have any DP or SA for more than 180 net days during the year before mBC diagnosis.

^b^ Adjusted ORs were calculated by adjusting crude ORs for age, calendar year of mBC diagnosis and SA net days in the year before mBC diagnosis (as continuous variables) and for the number of distant metastatic sites at mBC diagnosis and marital status (as categorical variables).

^c^ Synchronous is defined as primary mBC or the development of mBC less than 6 months after an early breast cancer diagnosis.

^d^ Could not be determined because of the multifocality of metastases.

^e^ Net days are calculated by multiplying the benefit received (i.e. 25%, 50%, 75% or 100%) by the total number of SA days benefit received.

### 3.3 Protective factors for the ability to RTW

The factors significantly associated with WND > 90 during the year after mBC diagnosis were synchronous mBC (AOR = 1.69, 95% CI 1.09–2.62), metachronous mBC within 24 months of primary BC diagnosis (AOR = 1.86, 95% CI 1.05–2.71), soft tissue, visceral, brain as first metastatic site (AOR = 1.47, 95% CI 1.05–2.06), and SA of 90 or fewer net days during the year before mBC diagnosis (AOR = 1.28, 95% CI 1.04–2.40) (Table 2). Age 50 years or younger and the number of metastases was not associated with WND > 90.

The factors significantly associated with WND > 180 were age 50 years or younger (AOR = 1.54, 95% CI 1.35–1.84), synchronous mBC (AOR = 1.67, 95% CI 1.04–2.68), metachronous mBC within 24 months of primary BC diagnosis (AOR = 1.51, 95% CI 1.24–2.34), and SA of 90 or fewer net days during the year before mBC diagnosis (AOR = 2.00, CI 1.52–2.72) (Table 2). The first site of metastasis and the number of metastases were not associated with WND > 180.

### 3.4 RTW by calendar year of metastatic BC diagnosis and other variables

**FIGURE 1:**
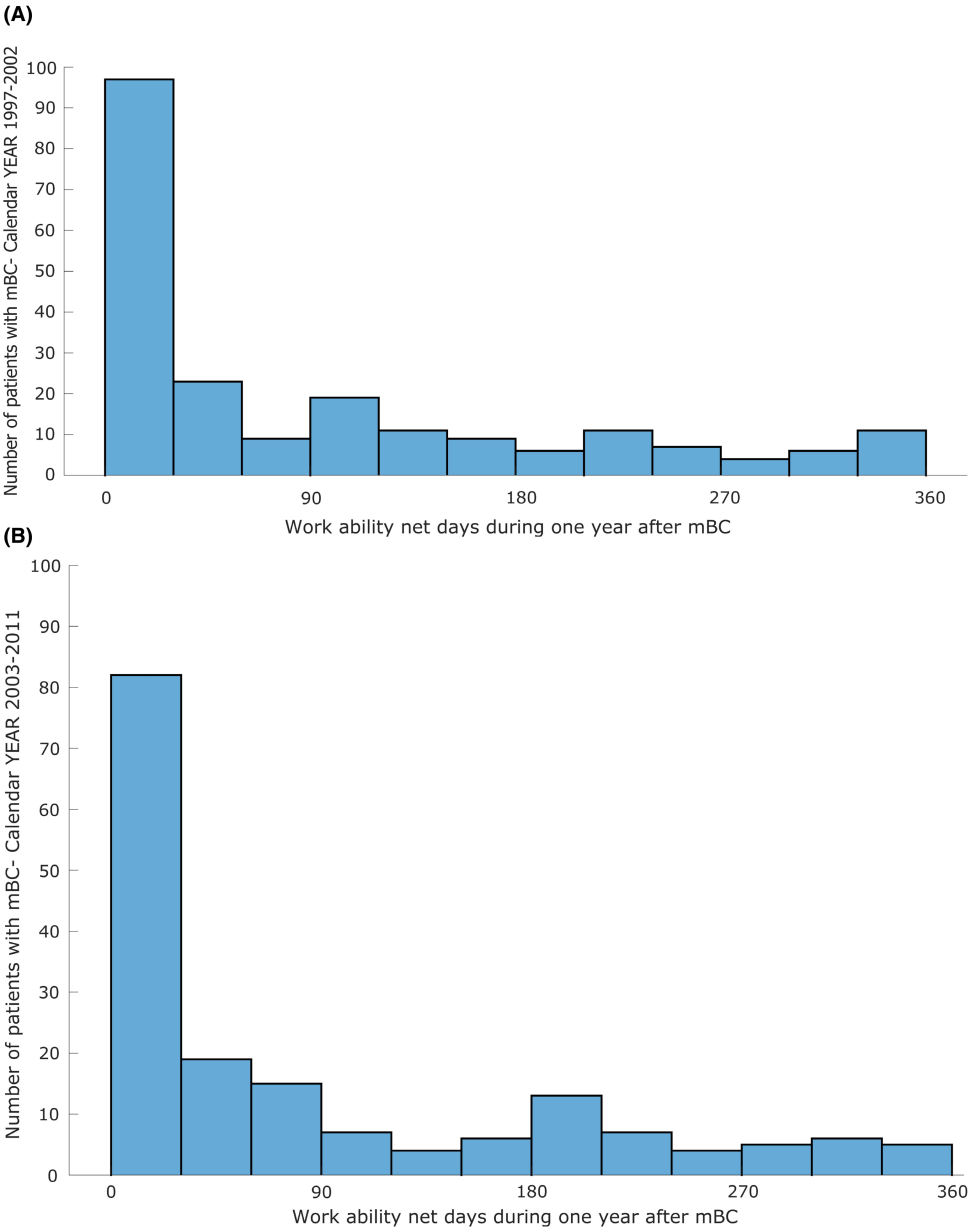
Distribution of 490 female patients with new mBC diagnosis in Stockholm‐Gotland Region, Sweden, January 1, 1997, to December 31, 2011, by WNDs during the year after mBC diagnosis. In the histograms, WNDs are divided into groups of 30 days, ranging from 0 to 30 days to 331 to 360 days. The histogram in (A) represents the 239 patients diagnosed with mBC between 1997 and 2002 (historical cohort) and in (B) represents the 251 patients diagnosed with mBC between 2003 and 2011 (current cohort, diagnosed after the introduction of contemporary oncological therapy). mBC, metastatic breast cancer; WND, working net day.

The mean (SD) WNDs was 134.9 (140.1) for patients diagnosed with mBC between 1997 and 2002 (historical cohort) and 161.3 (152.4) WNDs for patients diagnosed with mBC between 2003 and 2011 (current cohort), and these differed significantly (*p* = 0.046) (data not shown). In contrast, WNDs did not differ significantly between other variable groups, including age at mBC diagnosis (*p* = 0.55), metastasis‐free time interval (*p* = 0.31) and SA of 90 or fewer net days during the year before mBC diagnosis (*p* = 0.35) (data not shown). The prevalence of patients within each 30‐day WNDs category (ranging from 0 to 30 net days to 331 to 360 net days) was determined and is presented as histograms for the 1997 to 2002 cohort (Figure 1A) and the 2003 to 2011 cohort (Figure 1B). In historical and current cohorts, 98 and 82 women with mBC, respectively, had 0 to 30 WNDs.

### 3.5 Survival

**FIGURE 2:**
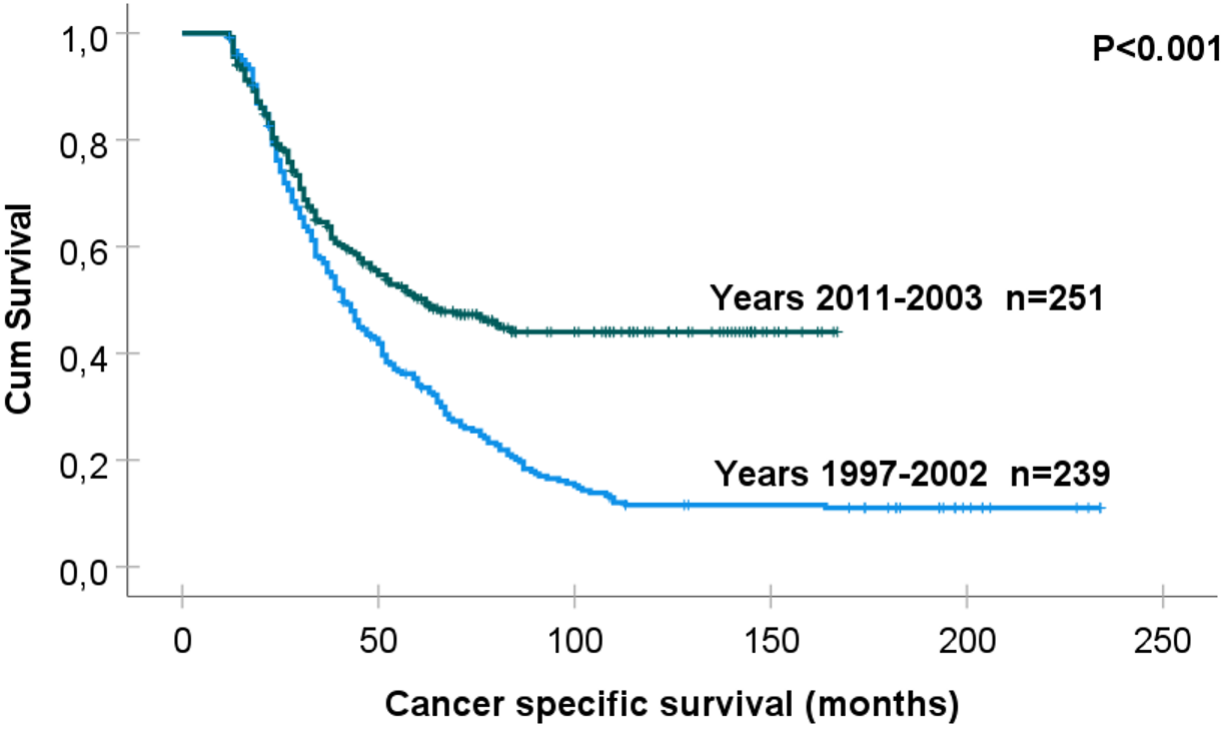
Kaplan–Meier curves demonstrate cancer‐specific survival in 490 female patients with new mBC diagnosis in Stockholm‐Gotland Region, Sweden, from January 1, 1997, to December 31, 2011. The mBC‐specific survival for patients diagnosed with mBC between 1997 and 2002 (historical cohort) was significantly shorter than for patients diagnosed with mBC between 2003 and 2011 (current cohort, diagnosed after the introduction of contemporary oncological therapy) (log‐rank test: *p* < 0.001). mBC, metastatic breast cancer.

The median (SE) mBC‐specific survival for patients diagnosed with mBC between 1997 and 2002 was 41.0 (2.5) months, and this was significantly shorter than the median (SE) mBC‐specific survival for patients diagnosed with mBC between 2003 and 2011, which was 62.0 (9.6) months (*p* < 0.001) (Figure 2).

## 4 DISCUSSION

Planning for RTW may be relevant for sub‐groups of patients with extended mBC survival. In this study, we analysed the prevalence of RTW of more than 25% (WND > 90) and 50% (WND > 180) during the year after patients received an mBC diagnosis, as well as whether specific demographic and tumour‐related factors were associated with RTW during that first year after mBC diagnosis. We found that 239 (48.8%) and 189 (38.6%) patients who lived a minimum of 1 year after the mBC diagnosis worked more than 90 net days and 180 net days, respectively, during the year after their metastatic diagnosis. During this year, partial RTW was significantly positively associated with ≤50 years of age at the time of mBC diagnosis, metastatic disease developing ≤24 months after eBC diagnosis, and <90 net days of sick leave taken during the year before mBC was diagnosed. The marital status and the number of children were not associated with RTW. Finally, we determined that patients diagnosed with mBC after 2003 had significantly more WNDs and better survival than those diagnosed before 2003, due to the introduction of contemporary oncology therapies.

Sesto et al. conducted an online survey study among 133 female respondents working at the time of mBC diagnosis; 72 were stably working, while 61 reported no‐longer‐working based on employment status at the time of the survey.^28^ According to Sesto et al., women living with mBC financial and/or insurance issues and the desire to support themselves and their families are reasons for considering work.^28^ A possible explanation for 180 (37%) women in our study having 0–30 WNDs could be due to the generous Swedish legislation against dismissal in the event of illness.

The landscape of mBC treatment changed considerably in 2003.^14^ Patients with mBC may be offered a wide range of therapeutic combinations, including various forms of systemic therapies and radiotherapy, with different durations of therapy. The treatment of mBC has been rapidly evolving, driven by an increasing understanding of the biological pathways underlying carcinogenesis, tumour growth and metastasis.^41^ For this study, we posited that including many different therapeutic agents and combinations as covariates might give rise to confusing or misleading results. Thus, we opted to categorise the treatment patients received by the calendar year their mBC was diagnosed; a historical cohort (diagnosed with mBC between 1997 and 2002) and a current cohort (diagnosed with mBC between 2003 and 2011). We considered that the categorisation approach might provide a more precise and fairer picture of the impact of treatment on RTW.

The patients in our study diagnosed with mBC in 2003 or later had significantly more WNDs during the first year after the diagnosis than patients diagnosed before 2003. This supports the introduction of more effective oncological agents and more options available to reduce the side effects of therapy. In particular, new systemic therapies that resulted in more extended periods of disease control and fewer adverse sequelae may have contributed to more WNDs observed in these patients.^11^, ^12^, ^13^ The fact that we observed no other significant differences in RTW within the other variables examined (i.e. age, metastasis‐free interval, SA during the year before mBC diagnosis) suggests that the differences in RTW between the historical and current cohorts were primarily attributable to the content of the therapies received by these patients.

Based on this study, younger age may be protective for having more WNDs during the year after mBC diagnosis. A likely explanation for the protective effect of younger age is that the patients may have been able to remain healthier than older patients after eBC diagnosis and subsequent mBC diagnosis. They may also have had less comorbidity before receiving the mBC diagnosis, been less vulnerable to the physical stresses caused by mBC, and been better able to tolerate subsequent treatment. We found that soft tissue, visceral and/or brain as the site of metastasis was associated with WND > 90, probably due to fewer symptoms and pain from the bone metastases.

It also appears that better health during the year before mBC diagnosis may be protective for having more WNDs during the year after mBC diagnosis. In this study, we used SA of 90 or fewer WNDs during the year before mBC diagnosis as a general proxy for having less/non‐comorbidity or few/non‐adverse sequelae after treatment for eBC.

We observed that compared with patients with 90 net days or less of SA during the year before mBC diagnosis, those with 91–180 net days of SA experienced a lower level of RTW. Our results are consistent with others who have reported that patients with illnesses and comorbidities tend to have reduced RTW.^34^, ^35^, ^36^, ^37^, ^38^, ^42^

### 4.1 Strengths and limitations

Our findings add depth to understanding factors contributing to RTW in patients diagnosed with mBC. To our knowledge, this is the first study of RTW done in a cohort of patients with mBC that has been based on national registers and has assessed the possible impact of tumour burden on RTW. Our use of data from high‐quality Swedish registers, which contain few dropouts or data gaps, suggests that the internal validity of this study is substantial.^35^, ^43^ This study's results are generalisable to female patients with mBC below the age of 63 years, in countries with similar mBC therapies, similar social security systems as in Sweden and comparable distributions of women in their workforces. Our findings may also be helpful for future studies of types of support and rehabilitation that might work best for women with mBC who desire to maintain or increase RTW after their diagnosis.

This study has several limitations. First, it does not provide information about how specific mBC therapies impact RTW. Second, our assessment of variables affecting RTW was mainly focused on clinical demographic and tumour‐related factors. However, we cannot rule out that factors other than those we studied may have also influenced RTW and our results.^44^, ^45^

### 4.2 Conclusions

Almost half of the women studied had RTW at least 90 WND in the first year after mBC diagnosis. Patients diagnosed with mBC in 2003 or later, after the introduction of contemporary oncological management for BC, had better survival and more WNDs than those diagnosed earlier. Planning for long‐term RTW for sub‐groups of mBC patients seems realistic. RTW was associated with being younger, developing metastases early and having limited comorbidities during the year before the diagnosis of mBC. Further studies are needed to determine the types of support and rehabilitation that might work best for women with mBC who desire to maintain their work ability.

## AUTHOR CONTRIBUTIONS

**Aina Johnsson:** Conceptualization (lead); methodology (equal); project administration (equal); writing – original draft (equal); writing – review and editing (equal). **Narsis A. Kiani:** Formal analysis (lead); writing – review and editing (equal). **Sofie A. M. Gernaat:** Data curation (supporting); methodology (supporting); writing – review and editing (supporting). **Ulla Wilking:** Data curation (lead); methodology (equal); writing – review and editing (equal). **Ivan Shabo:** Conceptualization (equal); formal analysis (supporting); methodology (equal); writing – review and editing (equal). **Elham Hedayati:** Conceptualization (lead); methodology (equal); project administration (equal); supervision (lead); writing – original draft (equal); writing – review and editing (equal).

## CONFLICT OF INTEREST STATEMENT

Financial interests: EH receives research funding from Roche and Pierre Fabre, all paid to Karolinska University Hospital. All remaining authors declare that they have no conflicts of interest.

## ETHICS APPROVAL

This study complied with the Declaration of Helsinki, and it was approved by the Regional Ethics Review Board at the Karolinska Institute (Dnr 2012/745–31). Permission was obtained to access and use the Breast Cancer Registry (BCR) and National Quality Register for Breast Cancer (NKBC) databases.

## CONSENT TO PARTICIPATE

Based on previous Swedish legislation, patients registered in the national quality registers do not need to provide written informed consent for their data to be included in healthcare research; however, they are informed that their data is included in registers and that they could opt out of that at any time.

## CONSENT TO PUBLISH

Based on previous Swedish legislation, patients registered in the national quality registers do not need to provide written informed consent for their data to be included in healthcare research and/or to be published.

## Supporting information


Table S1.

## Data Availability

The datasets generated and/or analysed during the current study are not publicly available because of Swedish law and regulations, but they are available from the corresponding author upon reasonable request.
