# Supplementary material for: Planning for return to work during the first year after breast cancer metastasis: A Swedish cohort study
Source: Cancer Med. 2023 Mar 6;12(9):10840–50. doi: 10.1002/cam4.5752 (PMC10225211; doi:10.1002/cam4.5752)
Supplement: Supplementary file 1 — Table S1. [file CAM4-12-10840-s001.docx]

**Supplementary Table 1.** International Classification of Diseases, Ninth Edition (ICD-9), codes used for categorising distant sites of metastases among 490 female patients with metastatic breast cancer (mBC) in Stockholm‐Gotland healthcare region, Sweden, January 1, 1997, through December 31, 2011

| **Sites of metastases** | **ICD-9 codes** |
| --- | --- |
| Bone-only | 198.5 Secondary malignant neoplasm of bone and bone marrow |
| Visceral non-brain | 197.0 Secondary malignant neoplasm of lung  197.2 Secondary malignant neoplasm of pleura  197.7 Malignant neoplasm of liver, secondary  198.4 Secondary malignant neoplasm of other parts of nervous system  195.1 Malignant neoplasm of thorax  195.2 Malignant neoplasm of abdomen  197.4 Secondary malignant neoplasm of small intestine including duodenum  197.5 Secondary malignant neoplasm of large intestine and rectum  197.6 Secondary malignant neoplasm of retroperitoneum and peritoneum  198.1 Secondary malignant neoplasm of other urinary organs  198.6 Secondary malignant neoplasm of ovary |
| Brain | 198.3 Secondary malignant neoplasm of brain and spinal cord |
| Non-visceral | 197.1 Secondary malignant neoplasm of mediastinum  196.1 Secondary and unspecified malignant neoplasm of intrathoracic lymph nodes  196.2 Secondary and unspecified malignant neoplasm of intra-abdominal lymph nodes  196.5 Secondary and unspecified malignant neoplasm of lymph nodes of inguinal region and lower limb  196.6 Secondary and unspecified malignant neoplasm of intrapelvic lymph nodes  196.8 Secondary and unspecified malignant neoplasm of lymph nodes of multiple sites  196.9 Secondary and unspecified malignant neoplasm of lymph nodes, site unspecified  198.2 Secondary malignant neoplasm of skin  196.0: Lymph nodes of head, face, and neck  196.3: Lymph nodes of axilla and upper limb |
